# Compliance of Healthcare Professionals with Safety Measures for Control of Hepatitis Viruses in Hemodialysis Centers: An Experience from Southeast Iran

**DOI:** 10.1155/2012/415841

**Published:** 2012-11-08

**Authors:** Sodaif Darvish Moghaddam, Mohammad Javad Zahedi, Mahdieh Dalili, Mostafa Shokoohi

**Affiliations:** ^1^Physiology Research Center, Department of Internal Medicine, Kerman University of Medical Sciences, Kerman 7616913911, Iran; ^2^Physiology Research Center, Afzalipour Hospital, Imam Exp, Kerman, Iran; ^3^Clinical Research Unit, Kerman University of Medical Sciences, Kerman, Iran; ^4^Research Center for Modeling in Health, Kerman University of Medical Sciences, Kerman, Iran

## Abstract

*Introduction.* Noncompliance with the recommended infection control measures by the healthcare professionals (HCPs) plays a major role in transmission of hepatitis B (HBV) and hepatitis C (HCV) viruses in hemodialysis (HD) wards. This study aimed to determine the compliance rate of the HCP with safety measures in the HD wards in southeast Iran. *Patients and Methods.* A total of 208 patients were enrolled. Adherence of HCPs with standard infection control measures was assessed. *Results. *Sixty-one HCPs with a mean age of 32.4 ± 11.2 years old were responsible for healthcare services. Compliance with the following items was weak: not sharing medications trolley (29.8%), disinfecting the shared instruments (46.2%), using single use materials for many patients (52.4%), carrying used materials in disposable containers (51.9%), not returning of unused materials to the clean room (55.3%), and adherence to hand washing (58.7%). Periodic monitoring for HBV and HCV was performed on 100% and 69.7% of the patients, respectively. Less than 2/3 of HCPs participated in the retraining courses. *Conclusion.* Compliance of HCPs with safety measures for viral hepatitis prevention was partly inadequate in HD wards. Emphasis on retraining of HCPs and official supervision would be effective steps in the reduction of viral dissemination.

## 1. Introduction

 The dissemination of hepatitis viruses among patients in hemodialysis (HD) centers is one of the most important causes of morbidity and mortality in end stage renal disease patients. While the introduction of vaccination programs and isolation of HD machines have limited the spread of HBV infection, its prevalence rates continue to be unacceptably high in most HD centers [1]. Prevalence of positive hepatitis B surface antigen (HBsAg) ranged between 1.3% and 14.6% in Asia-Pacific countries and 13.3% in Turkey [2, 3]. Hepatitis C virus (HCV) is also a major cause of liver disease in HD patients. HCV infections are usually asymptomatic and may be transmitted to others insidiously [4]. Prevalence of HCV in HD wards has been reported from 5% to 60% in different countries [5]. High risk behaviors and blood transfusion are not the usual routes of HCV transmission in HD patients. Environment of HD and failure to follow the safety measures for infection control may be the main cause of HCV dissemination in these centers [6]. Accordingly the kidney disease improving global outcome (KDIGO) in 2008 and the center for disease control and prevention (CDC) in 2001 advised protocols for infection control in HD centers. Principally, these recommendations are based on: (a) compliance with infection control protocols by healthcare professionals (HCP), (b) performing viral serological tests periodically, and (c) continuing training courses for personnel [7, 8]. Adherence to these recommendations eventually reduces and prevents new hepatitis viruses in HD centers. The main practical points to be considered are cleaning the rooms and patients' area, disinfection of instruments, correct drug preparation, and regular hand hygiene [9, 10]. Appropriate staff training and regular monitoring for hepatitis viruses are also mandatory [8]. In a study from Saudi Arabia, by utilization of these recommendations, no new case of HCV was found in a period of 2 years [11].

Viral hepatitis infections are still the challenging problem in HD centers in Iran. In a review by Alavian et al., the rate of HCV infection in HD patients was 5–24% in 2010 [12]. Serologic markers of HBV and HCV showed a declining trend from 3.8% and 14.4% to 2.6% and 4.5%, respectively, in recent years [13], but it varies in different parts of the country. In our recent report, seroprevalence of HCV, HBV, and HCV-HBV coinfection was 7%, 7%, and 1.7%, respectively, in HD centers in Kerman province [14]. Ministry of Health recommended universal anti-infective standard precautions to all HD centers but they are not supervised officially [13]. In order to clarify the contributing risk factors for higher viral transmission in this part of country, this study was conducted to determine the compliance rate of HCP with safety recommendations of KDIGO and CDC in southeast of Iran.

## 2. Patients and Methods

This cross-sectional study was carried out in seven HD centers around the whole province of Kerman, in 2011. Out of 208 HD patients, 91 cases were under healthcare services in Kerman city, the center of the province and the remained 117 cases were distributed in other 6 cities. The data were collected by using a check list based on the safety recommendations of CDC and KDIGO protocols. These recommendations are a major part of educational program both at the beginning of work and also during the annual retraining courses for HD personnel in Iran. The content of the check list comprised of three parts: (a) necessary care taken by the HCP (18 items), (b) periodic viral serological assessment (4 items), and (c) participation of HCP in retraining courses (4 items).

The first part of the study was observed during working hours. After the first part finished, the other two parts were assessed by interviewing with the HCP and reviewing the documents. We defined the rate of observance of recommended protocols by HCP into four categories: (a) excellent: ≥90%, (b) good: 80–89%, (c) adequate: 70–79%, and (d) weak: less than 69% compared to standard (100%) safety measures by CDC and KDIGO protocols. 

The relative and absolute frequency was used for presentation of the descriptive data. Chi-square and Fisher exact tests were used for data analysis. Data were analyzed using SPSS version15 (SPSS Inc., Chicago, IL, USA) software. *P* value less than 0.05 was considered significant.

## 3. Results

Out of 208 hemodialysis patients, 135 (64.9%) cases were males. Dialysis was performed in three running times: 138 (66.3%) cases in the morning, 58 (27.9%) cases in the evening, and 12 (5.8%) cases at the night hours. HCP who worked in dialysis wards were 61 persons (36 females, 25 males) with a mean age of 32.4 ± 11.2 years old. 

### 3.1. HCP Compliance with CDC-KDIGO Recommendations

The first item of this section was the presence of a dedicated clean room in the hemodialysis wards. All of the 7 HD wards had a clean room. The results of the remained 17 questions are shown in Table 1. The level of adherence of HCP was “adequate to excellent” in 10 items. “Weak adherence” was observed in 7 items: not sharing of trolley to carry patients' medications (29.8%), cleaning and disinfecting the shared instruments (46.2%), using single use materials for many patients (52.4%), carrying used materials in disposable and nonpermeable containers (51.9%), not returning of unused materials to the clean room (55.3%), adherence to adequate hand washing (58.7%) and not drawing drug for injection to many patients from a single vial (67.3%). No difference was found in level of care for male and female patients. A significant difference was observed for some of the items between working shifts (Table 1).

### 3.2. Monitoring of Viral Serological Markers

HBV monitoring including HBs Ag detection, HBV vaccination, and regular measurement of HBs antibody (Ab) titer had been performed in all of the patients.

HCV Ab test was performed once in every six months in 69.7% of patients. It covered 74.1% of men and 61.6% of women (*P* = 0.063). In case of persistent high alanine aminotransferase (ALT) level and negative HCV Ab, HCV PCR was measured in 2.4%. Compliance of HCP to report the new cases of positive HCV to the local CDC was 92%. Monitoring of viral markers had a significant difference in favor of “other cities” than Kerman, the center of the province (*P* < 0.001) (Table 2).

### 3.3. Participation of HCP in Annual Retraining Courses

**Figure 1:**
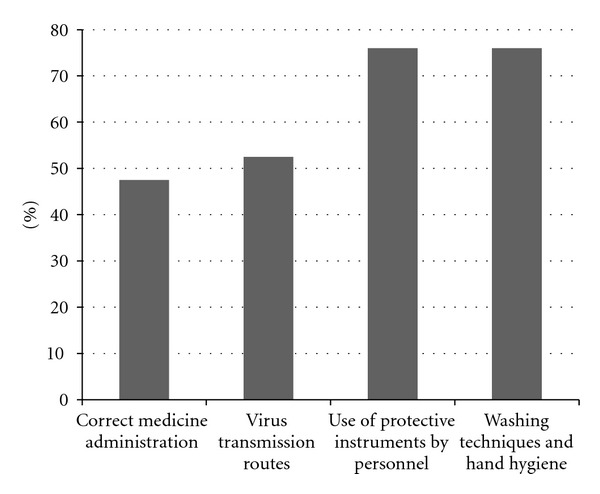
The rate of participation of personnel in annual retraining courses in HD wards.

The percentage of HCP who participated in the annual retraining courses were as hand hygiene practice, 76%; use of protective instruments by HCP, 76%; routes of dissemination of viruses, 52%; methods of correct administration of medications, 47% (Figure 1).

## 4. Discussion

The aim of this study was to assess the rate of compliance of HCP with safety measures for control of hepatitis viruses in HD wards in southeast Iran. Compliance of HCP with many items of KDIGO and CDC recommendations was adequate to excellent. The main noncompliant items were: sharing the medications trolleys, no disinfection of instruments, reuse of single use materials, return of unused materials to the clean room, and no adherence to hand hygiene.

HD associated viral hepatitis are a challenging health problem around the world and especially in developing countries such as Iran. Prevalence of HBV in Iranian general population declined to 2.6% due to public HBV vaccination and improved public health awareness in recent years [15]. HCV prevalence is low in Iranian general population and is estimated to be less than 1% [16]. On the other hand, prevalence of HCV and HBV in HD wards in Iran has been reported 5–24% and 7%, respectively [12, 14]. Potentially HD wards could be a source of hepatitis virus infections from now on. It seems that poor compliance with infection control measures and inadequate disinfection of HD equipments play a major role in hepatitis virus dissemination. 

In several studies HD environment was the most important factor for hepatitis virus transmission. It is highly recommended that medicines should be prepared in clean areas away from dialysis apparatus and served in separate trays for different patients. Multidoses vials should not be used and the drugs should be carried in separate trolleys for each patient [17, 18]. Based on a survey on HD centers from USA in 2002, HBV incidence was higher in centers with inappropriate drug preparation [19]. In our study, preparation and carrying medicines were served inadequately and they need to be revised by HD staffs.

HD wards equipments including bed, chair and external surfaces of the dialysis apparatus and instruments like scissors, stethoscopes, and blood pressure cuffs should be cleaned and in cases should be disinfected for every patient [20]. These items were also among the other “weak” adherent points in our study.

HCP may play a role in transmission of infections especially HCV in HD wards [20]. Hand hygiene practices are mandatory for prevention of viral spreading. In our study, wearing of gloves by personnel was excellent (98%) but they had a weak compliance rate with hand washing (58%). Similar results have indicated in other studies [18, 21, 22]. In Girou et al. study, the rate of compliance with hand hygiene was 37%, and gloves were immediately removed after patient care in 33% [18]. Shimokura et al. showed that the dialysis staff adhered to hand washing and changing gloves in 57% before starting dialysis, in 55% between injections of medications to different patients, and in 47% when changing from one patient to another [21].

Monitoring of viral markers is also essential for prevention of hepatitis viruses. In our study monitoring of HBV infection was satisfactory but it was weak for HCV, especially at the center of province. Better performance of HCP in “other cities” could be mainly due to low volume of patients and higher ratio of personnel to patients. According to CDC recommendations all HD patients should be screened and followed for HCV infection by anti-HCV, HCV PCR, and ALT level determination [8]. In the present study, most of the new cases were reported to the local CDC. Periodic test of anti-HCV was performed in 69% of patients, but monthly assessment of ALT and in case of high ALT, PCR request, were the main defect points. In the 2002 survey of US HD wards, 47% of facilities reported not performing routine anti-HCV screening [19].

Attention to the primary education of personnel and regular annual retraining courses are the main steps in the elevation of performance of personnel in hemodialysis centers. In a study from Sudan, although the knowledge of HCP about infection control measures in HD centers was good (81%) but their performance in patients' bed was weak (8–23%) [23]. In our study 47–76% of personnel have passed the required training courses, and it should be improved in future. 

## 5. Conclusion

Although there is a decreasing trend in the frequency of viral hepatitis in Iran, it seems that still HD patients have a higher risk of infections mainly due to ignoring of safety measures to minimize the risk of transmission. Emphasis on observance of standard infection control recommendations, retraining of HCP, and official supervision on performance of HD centers would be effective steps to reduce the viral transmission.

## Figures and Tables

**Table 1:** The rate of observance of protocols by HD personnel in working times per patients.

	Personnel Care	Shift time			
		Morning *n* = 138 (%)	Afternoon *n* = 58 (%)	Night *n* = 12 (%)	Total *n* = 208 (%)
(1)	Not drawing drug for injection to many patients from a single vial	94 (68.6)	34 (58.6)	12 (100)^¥^	140 (67.3)
(2)	Preparation of medicine for patients in clean room and far from dialysis stations	109 (79.6)	55 (94.8)	12 (100)^¥^	176 (84.6)
(3)	Not returning unused medicines to clean room	109 (79.6)	55 (94.8)	12 (100)^¥^	176 (84.6)
(4)	Carrying medicine separately for each patient from clean room	96 (69.6)	55 (94.8)	12 (100)^¥^	163 (78.4)
(5)	Not sharing trolley for carrying medicine for different patients	40 (29)	20 (34.5)	2 (16.7)	62 (29.8)^Ψ^
(6)	Using single use materials like adhesive plaster and alcohol swab for more than one patient	71 (51.4)	38 (65.5)	0 (0)^¥^	109 (52.4)^Ψ^
(7)	Not returning unused instruments to clean room	71 (51.4)	33 (56.9)	11 (91.7)^¥^	115 (55.3)^Ψ^
(8)	Carrying used materials in disposable and impermeable containers	69 (50)	37 (63.8)	2 (16.7)^¥^	108 (51.9)^Ψ^
(9)	Changing gloves by personnel when attending every patient	137 (99.3)	56 (98.2)	11 (91.7)	204 (98.1)
(10)	Washing hands by personnel when attending every patient	87 (63)	28 (48.3)	7 (58.3)	122 (58.7)^Ψ^
(11)	Covering guan, glasses, and mask by personnel	98 (71)	52 (89.7)	10 (83.3)^¥^	160 (76.9)
(12)	Cleaning and disinfecting the surfaces of the dialysis apparatus and patient bed for every patient	128 (93.4)	55 (94.8)	12 (100)	195 (93.8)
(13)	Appropriate time intervals for cleaning external surfaces before using by patients	136 (99.3)	55 (94.8)	12 (100)	203 (97.6)
(14)	Disinfecting shared instruments like blood pressure cuff, stethoscope, and scissors for each patient	59 (42.8)	37 (63.8)	0 (0)^¥^	96 (46.2)^Ψ^
(15)	Not eating, drinking, and smoking when attending patients	121 (87.7)	54 (93.1)	12 (100)	187 (89.9)
(16)	Cleaning and disinfecting when observing blood in places	125 (91.2)	57 (98.3)	12 (100)	194 (93.3)
(17)	Cleaning and disinfecting dialysis apparatus based on protocol regulations	129 (93.5)	54 (93.1)	12 (100)	195 (93.8)

^¥^Significant between working shifts, ^Ψ^weak compliance rate.

**Table 2:** HCV monitoring of HD patients according to working shifts and place of residence.

HCV monitoring	Total*	Sex		Shift			City	
	*N* = 208	Male 135	Female 73	Morning 138	Afternoon 58	Night 12	Kerman 91	Others 117
Checking HCVAb every six months	145 (69.7)	100 (74.1)	45 (61.6)	103 (74.6)	40 (69)	2 (1.7)	36 (39.6)	109 (93)
Monthly checking of serum ALT in HCVAb negatives	102 (49)	69 (51.1)	33 (45.2)	61 (44.2)	39 (67.2)	2 (1.7)	8 (8.8)	94 (80.3)
Conducting HCV PCR test in HCVAb negative patients with high ALT	5 (2.4)	3 (2.4)	2 (2.8)	3 (2.3)	2 (3.6)	0 (0)	1 (1.1)	4 (3.4)

*N (%).
